# Somatic Mutations During an Immune Response in *Xenopus* Tadpoles

**DOI:** 10.1155/1995/38639

**Published:** 1995

**Authors:** Melanie Wilson, Anne Marcuz, Louis Du Pasquier

**Affiliations:** 1 Department of Microbiology University of Mississippi Medical Center 2500 North State Street Jackson Mississippi 39216-4505 USA; 2 Basel Institute for Immunology Grenzacherstrasse 487 Basel CH-4005 Switzerland

**Keywords:** Antibody diversity, LG hybrid

## Abstract

The tadpole B-cell repertoire is less diverse than that of the adult frog; their antibodies are of lower affinity and are less heterogenous. In order to determine whether this difference is due to a lack of or a reduced rate of somatic hypermutation, we analyzed and compared cDNA sequences utilizing V_H_1 elements with germline counterparts in isogenic LG7 tadpoles during an immune response. Indeed, tadpole V_H_1 sequences contained somatic mutations. There were zeo to 5 mutations per sequence, all single base-point mutations, with the high ratio of GC to AT base-pair alterations similar to that observed in adult frogs.


*Developmental Immunology*, 1995, Vol 4, pp. 227–234
Reprints available directly from the publisher
Photocopying permitted by license only

© 1995 Harwood Academic Publishers GmbH
Printed in Singapore

### Somatic Mutations During an Immune Response in *Xenopus* Tadpoles

MELANIE WILSON,[‡+] ANNE MARCUZ,[‡] and LOUIS DU PASQUIER[‡*]

[+]*Department of Microbiology, University of Mississippi Medical Center, 2500 North State Street, Jackson, Mississippi 39216-4505*
[‡]*Basel Institute for Immunology, Grenzacherstrasse 487, CH-4005 Basel, Switzerland*

The tadpole B-cell repertoire is less diverse than that of the adult frog; their antibodies are of lower affinity and are less heterogenous. In order to determine whether this difference is due to a lack of or a reduced rate of somatic hypermutation, we analyzed and compared cDNA sequences utilizing $V_H1$ elements with germline counterparts in isogenic LG7 tadpoles during an immune response. Indeed, tadpole $V_H1$ sequences contained somatic mutations. There were zeo to 5 mutations per sequence, all single base-point mutations, with the high ratio of GC to AT base-pair alterations similar to that observed in adult frogs.

KEYWORDS: Antibody diversity, LG hybrid.

### INTRODUCTION

The African clawed frog, *Xenopus*, provides an attractive model for studying the ontogeny of the immune system and much is known about the changes that occur at metamorphosis (reviewed by Flajnik et al., 1987). *Xenopus* larval antibody responses are not influenced maternally and are clearly distinct from adult antibody responses. The tadpole B-cell repertoire is less diverse than that of the adult frog, which is considered more limited than that of mammals (Du Pasquier et al., 1989). Tadpole antibodies are of lower affinity than adult antibodies specific for the same antigen and their isoelectric focusing patterns of antigen-specific antibodies are less heterogeneous (Du Pasquier et al., 1979; Hsu and Du Pasquier, 1984a, 1984b). These differences are not correlated with the stage of differentiation, not size or age. Very large, very old tadpoles produced by blocking metamorphosis with sodium perchlorate (Du Pasquier et al., 1985) make only tadpole-type antibodies (Hsu and Du Pasquier, 1992).

*Xenopus* immunoglobulin genes are organized and rearranged in a way similar to that of mammals (Schwager et al., 1988a) with multiple $V_H$, multiple D ($\geq$ 16), multiple J (8–9), and a single constant region gene. Eleven $V_H$ families (Haire et al., 1990) with ca. 2 to 32 members per family (Wilson et al., 1992b) offer a large diversity of viable region genes.

In *Xenopus*, as in mammals (Tonegawa, 1983), CDR3s are the most diverse. Nucleotides may be deleted from and/or added to $V_HD$ and $DJ_H$ joints during rearrangement (Tonegawa, 1983; Lafaille et al., 1989). However, like in fetal and newborn mice (Feeney, 1990; Gu et al., 1990), *Xenopus* tadpoles lack N diversification, which might partially explain the low heterogeneity of tadpole antibodies (Schwager et al., 1991).

More recently, tadpole CDR3 were also found to be much shorter than adult CDR3 (Lee et al., 1993); although the differences in CDR3s might account for the observed lower heterogeneity of tadpole antibodies, they do not necessarily explain the lower affinity. Although we originally proposed that somatic mutation might play a reduced role in *Xenopus* antibody responses and thus limit the affinity maturation of *Xenopus* antibodies (Du Pasquier, 1982), our recent results suggest that this is not the case, at least in adult (Wilson et al., 1992a). The rate of somatic mutation in *Xenopus* adult is well within the range reported in mice (McKean et al., 1984; Wabl et al., 1985; Kocks and Rajewsky, 1989), but the mutants are apparently poorly selected. This situation could lead easily to the observed minimal affinity maturation of the antibody response of many cold-blooded vertebrates. Larvae have an even more restricted antibody repertoire and a

*Corresponding author.

response characterized by an affinity lower than in the adult, at least in the case of the response to dinitrophenol coupled to keyhole limpet hemocyanin (DNP-KLH) (Hsu and Du Pasquier, 1984a). It is therefore possible that the difference between adults and larvae could be due to the absence of somatic mutation in tadpoles. To investigate this point, we analyzed cDNA sequences from immunized LG7 tadpoles. LG7 is an isogenic line derived from an *X. laevis/X. gilli* hybrid (Kobel and Du Pasquier, 1975), which is homozygous at the immunoglobulin (Ig) heavy = (H) chain locus (Wilson et al., 1992b).

### MATERIALS AND METHODS

### Animals and cDNA Library Construction

Twelve isogenic LG7 tadpoles (*X. laevis/X. gilli;* Kobel and Du Pasquier, 1975) were blocked at stage 52 using sodium perchlorate, as previously described (Du Pasquier et al., 1985). After 6 months, the animals were hyperimmunized with DNP-KLH according to a protocol known to produce the best antibody responses in tadpoles (Hsu and Du Pasquier, 1984a). Four weeks after the last injection, 5 animals were sacrificed and about 1 μg mRNA was prepared from their pooled livers and spleens using a Fast Track mRNA kit (Invitrogen). LG7 tadpole cDNA was synthesized with a kit from Pharmacia and cloned into the lambda Zap II vector system from Stratagene. The unamplified library, which contained 1 x $10^7$ recombinant phage clones, was screened at high stringency with *Xenopus* Cμ, Cυ and $V_H1$, $V_H5$ probes (kindly provided by our collegue J. Schwager). The probes were labeled by random priming (Feinberg and Vogelstein, 1983) and hybridization conditions were as described by Wilson et al. (1986). Positive clones were plaque purified and subcloned into Bluescript according to the manufacturer's (Strategene) recommended protocol.

### Sequencing and Computer Analysis

The double-stranded recombinant plasmid cDNAs were sequenced on both strands by primer extension with the dideoxnucleotide triphosphate chain-termination-reaction method (Sanger et al., 1977). Synthetic oligonucleotides and universal primers were prepared by H.R. Kiefer's laboratory (Basel Institute for Immunology). We consider the error frequency of the modified T7 polymerase in our sequencing reactions to be negligible, because 2 independent Cμ cDNAs were sequenced (4830 bp each) with no errors.

All computer programs were written by C. Steinberg (Basel Institute for Immunology). DNA sequences were aligned in pairs with a program based on the algorithm of Needleman and Wuntsch (1970). Each sequence was aligned to a master, and the pairwise alignments were used as input for a multiple alignment based on a heuristic algorithm.

### RESULTS

### Analysis of Expressed $V_H1$ cDNAs

From the unamplified library, we isolated and sequenced 5 μ and 6 υ cDNAs containing $V_H1$ family elements. Only 1 of the μ cDNAs could be matched with a $V_H1$ germline sequence (Wilson et al., 1992b). The other 4 μ cDNAs were too short to be assigned; their sequences began in either CDR2 or FR3. In fact, many of the positive μ and υ cDNAs represented short transcripts (for example, only 21 of 73 υ positive cDNAs screened positive with a J consensus oligo).

All of the υ cDNAs had identifiable $V_H1$ germline gene counterparts. One of them (LG7t3) was unmutated and corresponded to the germline gene LG7G341.

Five (4 υ and 1 μ) out of the 7 analyzable $V_H1$ cDNAs form a related group, and they are shown in Fig. 1. cDNAs υt8 and υt4 are clearly derived from the GL7g21 genomic sequence. These 2 cDNAs are identical and presumably form a clone (clone 1), because both have identical $V_HD$ and $DJ_H$ joints. As in our previous studies, we assume that if 2 similar genes (similar CDRs 1 and 2, similar FRs) have identical leader sequences, they are likely to be derived from the same germline $V_H$ (Wilson et al., 1992a). Both υt8 and υt4 have a point mutation in their leader sequence; CTA becomes TTA. However, because of their close identity throughout the coding region to LG7g21 (only 2 point mutations), we match them with LG7g21. cDNAs μt14, υt12, and υt6 appear to be derived from a germline $V_H1$ related to LG7g21. These 3 cDNAs all rearrange to different $DJ_Hs$; Fig. 1 shows the differences that they share as compared to LG7g21. In this group, 8 nucleotide differences are scattered throughout the

```

          ---------1---------2---------3---------4---------5---------6

LG7g21    ATGAAGTTGTTTCTAGTTTGTTTATTGTTCCTCTCAGGTTTATCAGGGGTTCACTCTGAC

LG7t8υ    ''''''''''''T'''''''''''''''''''''''''''''''''''''''''''''''

LG7t4υ    --------''''T'''''''''''''''''''''''''''''''''''''''''''''''

LG7t14μ   '''''''''''''''''''''''''''''''''''''''''''''''''''''''''''T

LG7t12υ   '''''''''''''''''''''''''''''''''''''''''''''''''''''''''''T

LG7t6υ    '''''''''''''''''''''''''''''''''''''''''''''''''''''''''''T

          ---------1---------2---------3---------4---------5---------6

LG7g21    ATACAACTTGACCAGTCAGAGCCAGTGGTGATAAAGCCGGGAGGGTCTCACAAACTGTCC

LG7t8υ    ''''''''''''''''''''''''''''''''''''''''''''''''''''''''''''

LG7t4υ    ''''''''''''''''''''''''''''''''''''''''''''''''''''''''''''

LG7t14μ   ''''''''''''''''''''''''''''''''''''''''''''''''''''''''''''

LG7t12υ   ''''''''''''''''''''''''''''''''''''''''''''''''''''''''''''

LG7t6υ    ''''''''''''''''''''''''''''''''''''''''''''''''''''''''''''

                                     |    CDR1    |

          ---------1---------2---------3---------4---------5---------6

LG7g21    TGCACAGCCTCTGGCTTCACATTCAGTGGTTATTATATGCACTGGATTAGACAGGCTCCT

LG7t8υ    ''''''''''''''''''''''''''''''''''''''''''''''''''''''''''''

LG7t4υ    ''''''''''''''''''''''''''''''''''''''''''''''''''''''''''''

LG7t14μ   '''''''''''''''''''''''''''A'C'''''''''''''''G''''''''''''''

LG7t12υ   '''''''''''''''''''''''''''A'C'''''''''''''''G''''''''''''''

LG7t6υ    '''''''''''''''''''''''''''ACC'''''''''''''''G''''''''''''''

                                 |            CDR2                  |

          ---------1---------2---------3---------4---------5---------6

LG7g21    GGGAAAGGATTACAGTGGGTGTCTCGTATAAGCAGCGACGGGGTAGCACAGTCTATGCT

LG7t8υ    ''''''''''''''''''''''''''''''''''A'''''''''''''''''''''''''

LG7t4υ    ''''''''''''''''''''''''''''''''''A'''''''''''''''''''''''''

LG7t14μ   ''''''''''''''''''''A'''''''''''''''''''''T'''''''''''''''''

LG7t12υ   ''''''''''''''''''''A'''''''''''''''''''''T'''''''''''''''''

LG7t6υ    ''''''''''''''''''''A'''''''''''''C'''''''T'''''''''''''''''

          ---------1---------2---------3---------4---------5---------6

LG7g21    GATTCAGTTAAAGGAAGATTTACCATCTCCAGAGACAATAACAACAACAAGTTATATCTG

LG7t8υ    ''''''''''''''''''''''''''''''''''''''''''''''''''''''''''''

LG7t4υ    ''''''''''''''''''''''''''''''''''''''''''''''''''''''''''''

LG7t14μ   '''''''''''''''''''''''''''''''''''''''''T''''''''''''''''''

LG7t12υ   '''''''''''''''''''''''''''''''''''''''''T''''''''''''''''''

LG7t6υ    '''''''''''''''''''''''''''''''''''''''''T''''''''''''''''''

          ---------1---------2---------3---------4---------5-----

LG7g21    CAAATGAACAATCTACAAACTGAAGACGCTGCCGTGTATTACTGTGCTAGAGATG

LG7t8υ    '''''''''''''''''''''''''''''''''''''''''''''A'''''''''

LG7t4υ    '''''''''''''''''''''''''''''''''''''''''''''A'''''''''

LG7t14μ   '''''''''''''''''''''''''''''''''C''''''''''''''''-----

LG7t12υ   '''''''''''''''''''''''''''''''''C''''''''''''''''CA

LG7t6υ    '''''''''''''''''''''''''''''''''C''''''''''''''''CA

```

FIGURE 1. Alignment of tadpole cDNAs related to the *Xenopus* germline $V_H1$ gene LG7g21. The master genomic sequence begins at the initiation codon ATG and continues down to the heptamer of its RSS. The leader intron is spliced out for the alignment. The cDNA sequences also begin at the initiation codon or at the first sequenced nucleotide of their leader and continue down to their CDR3 boundaries. Sequence identities are indicated by primes (') and gaps (-) are introduced to maximize homology. The CDR boundaries are marked and are according to Kabat et al. (1991). The GenBank accession number for LG7g21 is M94841.

coding region and 2 of the cDNAs, υt12 and υt6, have a CA at their $DJ_H$ joints (see what follows). We have assigned these 2 nucleotides to the $V_H$, assuming that deletion is occurring at $V_HD$ in μt14, bringing the total of nucleotide differences to 10. In adult LG7 frogs (Wilson et al., 1992a), the average number of mutations per gene was 3, and because it is unlikely that 3 different cDNAs would contain 10 identical point mutations, we have assumed that this group was derived from a new germline gene, which we have designated $V_H$g21B. Thus, υt6 contains 2 mutations, as it differs from μt14 and υt12 by 2 nucleotides.

Finally, cDNA LG7t13 is a somatic mutant of the germline gene $V_H$g346B.

Table 1 is a summary of the mutations found in all of the $V_H$1 cDNAs, and Fig. 2 gives the alignments of the cDNAs where mutations are found. Only the codons that contain mutations are shown. The FR and CDR boundaries, which are marked, were identified according to Kabat et al. (1991). Overall, there are 11 point mutations in 4 cDNAs; no deletion mutations were observed. Six of the mutations occur in the CDRs, 3 in the FRs, and 2 in the leaders; all code for replacement changes. In addition, all of the mutated CDR codons are serines, and 4 of them create changes in charge—2 at codon 53 in $V_H$g21 and 1 at codons 52 and 53 in $V_H$g346B. All of the mutations involve an alteration at a GC base pair.

### Analysis of the Tadpole $V_H$1 CDR3s

The entire CDR3 sequences for all the tadpole $V_H$1 cDNAs are shown in Fig. 3. The sequences are arranged according to $V_H$1 usage and are separated into segments derived from the germline $V_H$, $J_H$; and putative D. The $J_H$ elements could be positively identified because the LG15 $J_H$ area has been mapped and sequenced (Schwager et al., 1991). $J_H$5 is used most frequently, followed by $J_H$3. All of the cDNAs, except for υt3, have shortened $J_H$s and lack 5 (υt8, υt4) to 7 (μt14) bases at their 5′ ends; these were deleted during rearrangement (Schwager et al., 1991) The 2 cDNAs of clone 1 contain a point mutation in $J_H$; TTC becomes TTT. This mutation is silent. No other mutations were observed in the $J_H$ segments. Two of the $V_{H1}$ sequences, including one switched to υ (υt6), were out of frame.

The *Xenopus* D cluster has yet to be mapped and only 1 D (D15) in the germline has been sequenced (clone G2; Schwager et al., 1991). However, on the basis of core sequence identities, 16 putative D elements have been deduced from cDNA sequences (Schwager et al., 1988a, 1988b; Hsu et al., 1989; wilson et al., 1992a). Five out of the 7 LG7 tadpole cDNAs have identifiable D core sequences (underlined in Fig. 3); and if assignment of D1 is correct for cDNAs υt8 and υt4, there is a point mutation in clone 1; GCT becomes CCT. This replacement change brings the total number of mutation to 15. Previously, we reported (Wilson et al., 1992a) that point mutations were rare in *Xenopus* CDR3s; only 1 putative point mutation in a D1 segment was found in a survey of 61 adult LG7 $V_H$1 cDNAs.

In addition, the LG7 tadpole sequences confirm the findings of Schwager et al. (1991) that *Xenopus* tadpole CDR3s are diversified by P nucleotide additions (Lafaille et al., 1989). Because the complete sequences through the recombination signal sequences (RSS) are known for the majority of the LG7 $V_H$1s and for the $J_H$s, P nucleotides can be recognized. For example, no nucleotides are deleted from the 3′ end of the cDNAs corresponding to $V_H$g21, $V_H$g346B, and $V_H$g341 nor are there any nucleotides deleted from the 5′ end of $J_H$4 in t3. Thus, the C at the $V_HD$ junctions of clone 1, complementing the G of $V_H$g21, can be a P nucleotide. Likewise, the C at the $V_HD$ joint and the A at the $DJ_H$ joint of t3 can be attributed to P. Because of the lack of germline D sequences, the presence of N diversity is more difficult to detect; however, based on the core identity of D5, N nucleotides may be present at the $V_HD$ junctions of μt14.

TABLE 1

Summary of Mutations

| $V_H$ | Total cDNAs | Point mutations | Base changes |
|---|---|---|---|
| g21 | 2 | 6 | 2C→T, 4G→A |
| g21B | 3 | 2 | 2G→C |
| g346 | 1 | 3 | 1C→G, 1C→A, 1G→C |
| g341 | 1 | 0 | |

### *Tadpole $J_H$ Usage*

Taking into account all the $V_H$ sequences (21) available from this tadpole library screening, we found that the $J_H$ usage preference was the same as in adult, namely, a strong preference for $J_H$3 usage could be detected (Fig. 4).

| | L | CDR2 | FR3 | |
|---|---|---|---|---|
| | -15 | 53 | 97 | |
| | **L** | **S** | **A** | |
| $V_Hg21$ | C T A | A G C | G C T | |
| | **F** | **N** | **T** | |
| υt8 | T ' ' | ' A ' | A ' ' | Clone 1 |
| | **F** | **N** | **T** | |
| υt4 | T ' ' | ' A ' | A ' ' | |

| | CDR1 | CDR2 |
|---|---|---|
| | 31 | 53 |
| | **S** | **S** |
| $V_Hg21B$ | A G C | A G C |
| | **T** | **T** |
| υt6 | ' C ' | ' C ' |

| | FR1 | CDR2 | CDR2 |
|---|---|---|---|
| | 30 | 52 | 53 |
| | **T** | **S** | **S** |
| $V_Hg346B$ | A C T | A G C | A G C |
| | **S** | **N** | **R** |
| υt13 | ' G ' | ' A ' | ' ' A |

FIGURE 2. Tadpole cDNA codons in which mutations are found. Sequences are aligned under the corresponding germline $V_H1$ sequence; only codons in which mutations occur are shown. The name and isotype of each cDNA; υ, or μ is listed in the left margin. The one-letter code for the encoded amino acid is shown in bold type above the codon. Numbers correspond to codon positions according to Kabat et al. (1991). The primes (') indicate sequence identity. The vertical lines flank the two identical $V_H$ cDNAs that have identical $DJ_H$ rearrangements. These two cDNAs are clone 1 because they probably are derived from a single rearrangement event at IgH. The GenBank accession number for $V_Hg346B$ is M94845.

### Comparison with Adults

$V_Hg21$ was one of the most widely used $V_H1$ members in the adult anti-DNP response studied earlier (Wilson et al., 1992a). One of the mutations, reported in larval υt8 and 4, was identical (position 53 in CDR2 G→A), and the D usage ($D_H1$, $D_H16$) could also be considered similar, although the reading frames were different. $D_H5$ used in one gene (μt14) was in the same reading frame as $D_H5$ used in another adult $V_H1$ cDNA using the $V_Hg$ 44 germline gene segment. $V_Hg346b$, also found in tadpole cDNA υt13, was used in adult (6 clones), but all the mutations were different, and there were no similarities in the D region. $V_Hg341$ was expressed without mutation in tadpole (υt3), as it was in adult. Altogether, the $V_H1$ member usage found in immune adults and immune tadpoles is similar; the same subset is used in adults and tadpoles. This predominance of certain $V_H1$ members among cDNA obtained in two different libraries made from immune animals together with the discovery of one identical mutation in CDR2 of tadpole and adult genes could indicate where selection takes place.

### DISCUSSION

Somatic mutation in the immunoglobulin genes of a cold-blooded vertebrate was first described in the amphibian *Xenopus* (Wilson et al., 1992a). It was concluded that antibody diversity in such species as amphibians and probably fish was not limited by the availability of mutants, but the ability to select them properly. The cited work left open the possibility that during ontogeny of the immune systems, the ability to produce somatic mutants appeared after metamorphosis. The present work demonstrates that this is not the case. Tadpole B cells are

| | $V_H$ | D plus N and P nucleotides | $J_H$ | | |
|---|---|---|---|---|---|
| $V_Hg21$ | | ↓ | | | |
| υt8 | ACT AGA GAT G | CG TAC CCT AGC GGG | TTT GAC TAC | D1 | $J_H3$ I Clone |
| υt4 | ACT AGA GAT G | CG TAC CCT AGC GGG | TTT GAC TAC | D1 | $J_H3$ I 1 |
| $V_Hg21B$ | | | | | |
| μt14 | GCT AG | G GCT TAC TGG GGT GGG AGC | G TAT TTC GAG CAC | D5 | $J_H8$ out of frame |
| υt12 | GCT AGA CA | A TGG GGT AGC AGG | GCT TTC GAT TAC | D16 | $J_H5$ |
| υt6 | GCT AGA CA | C CTA GCG GGT ACA GGG GG | GCT TTC GAT TAC | D1 | $J_H5$ out of frame |
| $V_Hg346B$ | | | | | |
| υt13 | GCT AGA GA | G GGG GGG | GCT TTC GAT TAC | -- | $J_H5$ |
| $V_Hg341$ | | | | | |
| υt3 | GCT ACA GAA G | CC CA | T GTC TAT CAC | -- | $J_H4$ |

FIGURE 3. The CDR3 sequences of the tadpole $V_H1$ cDNAs grouped according to the germline $V_H$ used (Wilson et al., 1992b). The codons between the invariant cysteine that marks the end of FR3 and the invariant tryptophan that marks the beginning of FR4 are grouped according to their derivation: $V_H$ (left), D (middle), and $J_H$ segments (right). Identifiable D nucleotides are underlined and the names of the D and $J_H$ segments (according to Schwager et al., 1991; Wilson et al., 1992a) are listed in the right margin. The name of the germline $V_H$ gene counterpart is at the top of each group. Point mutations in clone 1 are double underlined. The GenBank accession number for $V_H341$ is M94843.

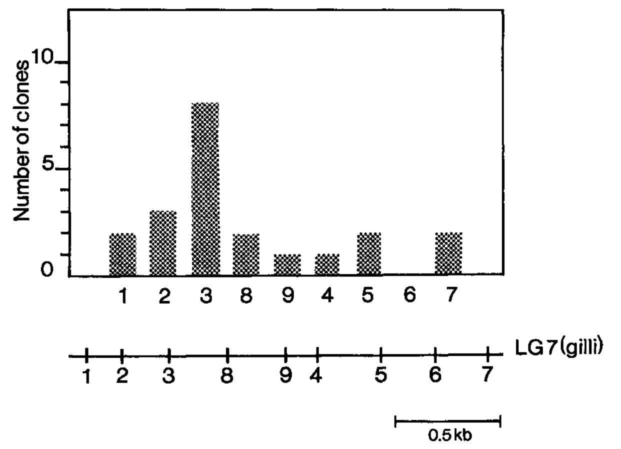

FIGURE 4. $J_H$ usage in tadpole VDJC rearrangements in LG7. The numbers on the abscissa refer to the $J_H$ number. The relative position of the $J_H$ on the chromosome segment is given underneath (from Schwager et al., 1991).

able to produce mutations that have the same characteristics as those of adults. Being limited in cell numbers when working with tadpoles, we cannot present any meaningful analysis of the R/S ratio or the position of the mutations within or outside the CDRs. However, all the bases altered by mutations were G or C, reflecting the same trend as the one observed in adults. The average number of mutations per V gene was of the same order of magnitude as in adults—1.5 in tadpole, 3 in adult. We therefore assume that the conditions of selection are also poor in tadpoles (like adults, tadpoles do not have bona fide germinal centers). The occurrence of somatic mutations in larvae raises an interesting question with respect to metamorphosis. In principle, there ought to be some mutants directed against future adult specificities. These would develop unnoticed and create autoimmune disorders when the corresponding epitope is generated. In this context, the poor ability to select mutants is perhaps advantageous. Being rarely selected, the potentially harmful mutant may not in fact be very dangerous.

Yet, as in adults, there must be some selection. Without clonal selection, no specific immune response would be noticeable. The finding of identical replacing mutations in adult and tadpole is also an indication that some gene products were selected. On the other hand, the similarity in D usage found in adult or tadpole without the conservation of the reading frame does not suggest a very strong selection by the antigen on the part of the antibody coded by this region. That some selection has taken place at the T-cell level is also shown by the fact that $V_H1$ rearrangements have switched, a highly T-cell-dependent event, to the second isotype IgY, even in tadpoles where this switch is rather poor (Hsu and Du Pasquier, 1984a, 1984b). Other $V_H$s analyzed from this library, picked at random by cross-hybridization with a $V_H5$ probe, were all μ, and, when comparison with germline genes was possible, unmutated. As a result of the present work, it is more difficult to explain the difference between larvae and adult antibody responses. However, the difference in T-cell help (Hsu and Du Pasquier, 1984a, 1984b) might play a role in the development of the response. The tadpole response would be measured at a stage corresponding to an early adult response. Another factor that could play a role is $V_H$ usage. Indeed, two $V_H1$ members prominently used in the adult response, $V_Hg$ 44a and $V_Hg$ 27, were not used in tadpoles of the same genetic background.

Several other interesting points arise from this analysis of the immunized tadpole cDNA library. There is the same type of bias in $J_H3$ usage in adults and tadpoles. Some $V_H$ seem not to be used at this stage of development. For instance, 23 cDNAs were isolated by cross-hybridization with a $V_H5$ probe, but after sequencing, none turned out to be $V_H5$, an observation consistent with previous work on tadpole Ig sequences, where no $V_H5$ usage was detected. The tadpole sequences presented here show as already observed (Schwager et al., 1991) that N diversity is very limited at this stage of ontogeny.

Finally, the observations that tadpole B cells can achieve somatic hypermutation and do not express CD5 (J̆urgens et al., in press, 1995) does not fit well with the notion that laryal B cells correspond to the Ly-1 B cells (now called B-1 cells; Kantor, 1991) of mammals, which is considered to be a primitive cell type (Herzenberg and Herzenberg, 1989).

### ACKNOWLEDGMENTS

We thank R. Mussman, J. Robert, and C. Steinberg for critical reading of the manuscript. The Basel Institute for Immunology was founded and is supported by Hoffman La-Roche, Basel, Switzerland.

*(Received February 16, 1995)*

*(Accepted May 5, 1995)*

### REFERENCES

Du Pasquier L. (1982). Antibody diversity in lower vertebrates. Why is it so restricted? Nature **296**: 311–313.

Du Pasquier L., Blomberg B., and Bernard C.C.A. (1979). Ontogeny of immunity in amphibians: Changes in antibody repertoires and appearance of adult major histocompatibility antigens in *Xenopus.* Eur. J. Immunol. **9**: 900–906.

Du Pasquier L., Flajnik M.F., Hsu E., and Guiet C. (1985). Methods used to study the immune system of *Xenopus* (Amphibian, Anura). *In:* Immunological methods, Lefkovits I., and Pernis B., Eds. (Orlando, FL: Academic Press), pp. 425–465.

Du Pasquier L., Schwager J., and Flajnik M.F. (1989). The immune system of *Xenopus.* Ann. Rev. Immunol. **7**: 251–275.

Feeney A.J. (1990). Lack of N regions in fetal and newborn mouse immunoglobulin V-D-J junctional sequences. J. Exp. Med. **172**: 1377–1390.

Feinberg A.P., and Vogelstein B. (1983). A technique for radiolabelling DNA restriction fragments to high specific activity. Anal. Biochem. **132**: 6–13.

Flajnik M.F., Hsu E., Kaufman J.F., and Du Pasquier L. (1987). Changes in the immune system during metamorphosis of *Xenopus.* Immunol. Today **8**: 58–64.

Gu H., Förster I., and Rajewsky K. (1990). Sequence homologies, N sequence insertion and $J_H$ gene utilization in $V_HDJ_H$ joining: Implications for the joining mechanism and the ontogenic timing of Ly1 B cell and B-CLL progenitor generation. *EMBO J.* **9**: 2133–2140.

Haire R.N., Amemiya C.T., Suzuki D., and Litman G.W. (1990). Eleven distinct $V_H$ gene families and additional patterns of sequence variation suggest a high degree of immunoglobulin gene complexity in a lower vertebrate, *Xenopus laevis.* J. Exp Med. **171**: 1721–1737.

Herzenberg L.A., and Herzenberg L.A. (1989). Toward a layered immune system. Cell **59**: 953–954.

Hsu E., and Du Pasquier L. (1984a). Ontogeny of the immune system in *Xenopus.* II. Antibody repertoire differences between larvae and adults. Differentiation **28**: 116–122.

Hsu E., and Du Pasquier L. (1984b). Ontogeny of the immune system in *Xenopus.* I. Larval immune response. Differentiation **28**: 109–115.

Hsu E., and Du Pasquier L. (1992). Changes in the amphibian antibody repertoire are correlated with metamorphosis and not with age or size. Dev. Immunol. **2**: 1–6.

Hsu E., Schwager J., and Alt F.w. (1989). Evolution of immunoglobulin genes: $V_H$ families in the amphibian *Xenopus.* Proc. Natl. Acad. Sci. USA **86**: 8010–8014.

Jürgens J.B., Gartland L.a., Du Pasquier L., Horton J.D., and Cooper M.D. (in press, 1995). Identification of a candidate CD5 homologue in the amphibian *Xenopus laevis.* J. Immunol.

Kabat E.A., Wu T.T., Reid-Miller M., Perry H.M., and Gottesman K.S. (1991). Sequences of proteins of immunological interest. (Bethesda, MD: National Institute of Health).

Kantor A.B. (1991). The development and repertoire of B-1 cells (CD5 B cells). Immunol. Today **12**: 389–391.

Kobel H.R., and Du Pasquier L. (1975). Production of large clones of histocompatible fully identical clawed toads (*Xenopus*). Immunogenetics **2**: 87–91.

Kocks C., and Rajewsky K. (1989). Stable expression and somatic hypermutation of antibody V regions in B-cell developmental pathways. Ann. Rev. Immunol. **7**: 537–559.

Lafaille J.J., Decloux A., Bonneville M., Takagaki Y., and Tonegawa S. (1989). Junctional sequences of T cell receptor genes: Implications for T cell lineages and for a novel intermediate of V-(D)-J joining. Cell **59**: 859–870.

Lee A., Desravines S., and Hsu E. (1993). IgH diversity in an individual with only one million B lymphocytes. Dev. Immunol. **3**: 211–222.

McKean D., Hüppi K., Bell M., Gerhard L., and Weigert W. (1984). Generation of antibody diversity in the immune response of BALB/c mice to influenza virus hemagglutinin. Proc. Natl. Acad. Sci. USA **81**: 3180–3184.

Sanger F., Nicklen S., and Coulson A.R. (1977). DNA sequencing with chain terminating inhibitors. Proc. Natl. Acad. Sci. USA **74**: 5463–5467.

Schwager J., Bürckert N., Courtet M., and Du Pasquier L. (1991). The ontogeny of diversification at the immunoglobulin heavy chain locus in *Xenopus*. Embo J. **10**: 2461–2470.

Schwager J., Grossberger D., and Du Pasquier L. (1988a). Organization and rearrangement of immunoglobulin M genes in the amphibian *Xenopus*. Embo J. **7**: 2409–2415.

Schwager J., Mikoryak C.A., and Steiner L.A. (1988b). Amino acid sequence of heavy chains from *Xenopus laevis* IgM deduced from cDNA sequences: Implication for evolution of immunoglobulin domains. Proc. Natl. Acad. Sci. USA **85**: 2245–2249.

Tonegawa S. (1983). Somatic generation of antibody diversity. Nature **302**: 575–581.

Wabl M., Burrows P.D., von Gabain A., and Steinberg C. (1985). Hypermutation at the immunoglobulin heavy chain locus in a pre B-cell line. Proc. Natl. Acad. Sci. USA **82**: 489–482.

Wilson M., Hsu E., Marcuz A., Courtet M., Du Pasquier L., and Steinberg C. (1992a). What limits affinity maturation of antibodies in *Xenopus* – the rate of somatic mutation or the ability to select mutants? EMBO J. **11**: 4337–4347.

Wilson M., Marcuz A., Courtet M., and Du Pasquier L. (1992b). Sequences of Cμ and the $V_H1$ family in LG7, a clonable strain of *Xenopus*, homozygous for the immunoglobulin loci. Dev. Immunol. **3**: 13–24.

Wilson M., Middleton D., Alford C., Sullivan J.T., Litman G.W., and Warr G.W. (1986). Putative immunoglobulin $V_H$ genes of the goldfish, *Carassius auratus*, detected by heterologous cross-hybridization with a murine $V_H$ probe. Vet. Immunol. Immunopath. **12**: 21–28.

